# Extraction of bioactive compounds from *Garcinia mangostana* L., using green technologies: a comprehensive analysis

**DOI:** 10.1007/s13197-025-06432-7

**Published:** 2025-09-09

**Authors:** Yurledys Torres Ortega, Somaris E. Quintana, Luis Alberto García-Zapateiro

**Affiliations:** 1 Research Group of Complex Fluid Engineering and Food Rheology, University of Cartagena, 130015 Cartagena, Colombia; 2 Unit Operations Department. Faculty of Engineering, Complex Fluid Engineering and Food Rheology Research Group (IFCRA), University of Cartagena, 130015, Cartagena de Indias, Colombia

**Keywords:** Biological activity, Bioactive compounds, *Garcinia mangostana*, Extraction technology, Xanthones

## Abstract

*Garcinia mangostana *L*.* is a tropical fruit celebrated for its substantial health benefits, largely due to its rich content of bioactive compounds. This review provides a detailed and current examination of various extraction technologies used to isolate these beneficial compounds and evaluates their associated biological activities. The fruit is particularly rich in phenolic compounds, anthocyanins, flavonoids, and isoprenylated xanthones, all of which are known for their potent antioxidant and anti-inflammatory properties. Traditional extraction methods, including solvent extraction and steam distillation, have been commonly employed to derive oils and their essential oils. However, these methods often involve lengthy extraction periods and significant solvent use, raising concerns about sustainability and efficiency. Conversely, modern extraction technologies such as supercritical fluid extraction, microwave-assisted extraction, and ultrasonic-assisted extraction offer enhanced efficiency, with benefits like reduced solvent use, shorter processing times, and higher yields. This review highlights recent advancements in these extraction methodologies, compares their effectiveness, and explores the potential applications of mangosteen-derived bioactive compounds across the food, pharmaceutical, and cosmetic industries. By evaluating both traditional and innovative techniques, this review aims to provide valuable insights into optimizing the extraction of mangosteen’s bioactive constituents for improved health benefits and industry applications.

## Introduction

*Garcinia mangostana* L., commonly known as mangosteen, is a tropical evergreen tree endemic to Southeast Asia, with significant cultivation in countries such as Malaysia, Thailand, Indonesia, Sri Lanka, the Philippines, Myanmar, and India (Ibrahim et al. 2016). This fruit-bearing tree belongs to the *Clusiaceae* family and the genus *Garcinia*, and it’s known as "Queen of Fruits" due to its distinctive sweet and tangy flavor, unique texture, and aromatic profile. Beyond its culinary appeal, mangosteen has garnered attention for its medicinal properties, which include antioxidant, anti-inflammatory, and antimicrobial effects (Aizat et al. 2019a). The fruit's rich composition of bioactive compounds, including xanthones, flavonoids, and polyphenols, contributes to its health-promoting benefits. The growing interest in mangosteen is not only due to its taste but also its potential applications in health and wellness industries, underscoring its value both as a food product and a therapeutic agent.

**Fig. 1:**
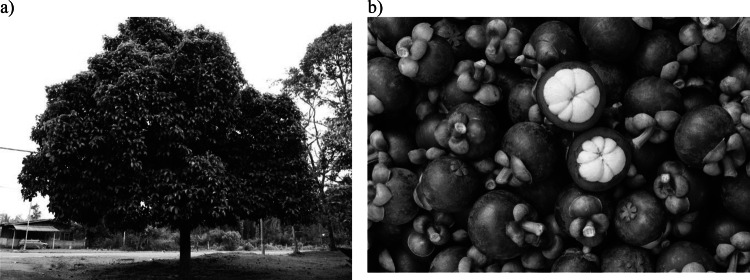
**a** Mangosteen tree and **b** Mangosteen fruits. Picture taken from website: https://sembramos.com.co/arbol-de-mangostino.html

The mangosteen tree is a robust evergreen species, reaching heights ranging from 6 to 25 m. It is distinguished by its thick, leathery leaves that provide a lush canopy, contributing to the tree's vibrant appearance (Aizat et al. 2019b). The fruit is round and encased in a thick, purple rind, which is visually striking and protects the delicate edible portion inside (Abdul-Rahman et al. 2017), as shown in Fig. 1. Within the fruit, the edible part is comprised of three to eight white arils, which are succulent and sweet-tasting (Saraswathy et al. 2022). The fruit is anatomically divided into approximately 17% external pericarp, 48% internal pericarp, 40% pulp, and 4% cap. Notably, over 60% of the fruit's weight is attributed to the pericarp, which is often considered waste biomass (Wang et al. 2018). This significant proportion of the fruit is typically discarded during processing, yet it holds potential value due to its rich content of bioactive compounds. Utilization of the pericarp could enhance the overall value of mangosteen by-products, offering opportunities for sustainable practices and the development of novel products.

*Garcinia mangostana* L., traditionally used in medicinal practices, has been widely employed to treat various ailments, including inflammatory diseases, skin infections, wounds, and ulcers (Oh et al. 2020). Over recent years, the medicinal properties of mangosteen have garnered increased attention from researchers, primarily due to its rich content of bioactive compounds. These compounds include phenolic acids, anthocyanins, prenylated xanthone derivatives, procyanidins, flavonoids, and tannins (Aizat et al. 2019b), all of which contribute to its therapeutic potential.

Notably, the majority of these beneficial compounds are found in the fruit's pericarp, the thick outer rind, which has been reported to exhibit antioxidant activity 20 times higher than that of the fruit’s edible flesh. In addition, the pericarp contains 10 times more phenolic compounds compared to the pulp, further enhancing its health benefits (Oh et al. 2020). This high concentration of bioactive compounds has been linked to a broad range of biological and pharmacological activities.

Several studies have demonstrated that mangosteen possesses a variety of therapeutic properties, including antioxidant, anti-obesity, antihyperglycemic, antidiabetic, antimicrobial, hepatoprotective, cardioprotective, anti-inflammatory, anti-Alzheimer, and pro-apoptotic effects (Mello et al. 2021). These findings suggest that mangosteen not only supports overall health but may also play a significant role in disease prevention and management. As the scientific community continues to explore the pharmacological potential of natural products, mangosteen stands out as a promising candidate for further investigation. Its rich composition of bioactive compounds and wide range of health benefits positions it as a potential natural remedy for numerous chronic conditions. Continued research is essential to fully understand the mechanisms behind its therapeutic effects and to determine the best methods for harnessing its medicinal properties in modern healthcare.

In recent years, there has been a significant rise in interest regarding the extraction techniques used to isolate bioactive compounds from *Garcinia mangostana* L. (Mohamed et al. 2017). Traditionally, conventional extraction methods have involved solid–liquid extraction using organic solvents such as methanol, ethanol, chloroform, ethyl acetate, acetone, and hexane. However, these methods present several disadvantages. First, the solvents employed in these processes are not only harmful to human health but also pose significant environmental risks. Additionally, conventional extraction methods often yield products with low purity due to the high concentrations of residual solvents. Other drawbacks include prolonged extraction times, low extraction efficiency, and the large quantities of solvents required (Chhouk et al. 2016). To address these limitations, several advanced extraction techniques—referred to as emerging technologies—have been explored in recent studies (Mohamed et al. 2017). These innovative methods aim to enhance the efficiency, safety, and environmental sustainability of extracting mangosteen’s valuable bioactive compounds. Techniques such as ultrasound-assisted extraction (UAE), microwave-assisted extraction (MAE), supercritical fluid extraction (SFE), and pressurized liquid extraction (PLE) have been investigated for their potential to improve extraction yields while reducing solvent use, extraction time, and environmental impact. The growing body of research on these emerging technologies highlights their ability to overcome the limitations of conventional methods. These advanced techniques are not only more efficient but also safer, producing higher-purity extracts that retain the full spectrum of bioactive compounds found in mangosteen. Given the potential health benefits of these compounds, particularly their antioxidant, anti-inflammatory, and antimicrobial properties, optimizing the extraction process is crucial for maximizing the therapeutic applications of mangosteen.

This review aims to provide an up-to-date overview of the various extraction technologies for isolating bioactive compounds from mangosteen. In doing so, it underscores the critical importance of these compounds in biological activity and highlights the progress being made in refining extraction methods to support future applications in nutraceuticals, pharmaceuticals, and functional foods.

## Bioactive compounds and biological activities

*Garcinia mangostana *L*.* is a valuable source of bioactive compounds present in various parts of the fruit, such as the pericarp, exocarp, seeds, and aril, which contain a wide range of phytochemicals, including phenolic acids, flavonoids, anthocyanins, tannins, and isoprenylated xanthones (Aizat et al. 2019b). The pericarp, accounting for approximately 70–75% of the bioactive components, is particularly rich in these compounds. The fruit is composed of up to 160 aromatic compounds in the epicarp and 105 in the endocarp, with at least 68 identified xanthones, among which α-mangostin, ß-mangostin, γ-mangostin, garcinone E, and gartanine are the most prominent, with α-mangostin and ß-mangostin being the most abundant in the pericarp (Mahmudah et al. 2020).

Xanthones, particularly α-mangostin, have been extensively studied due to their diverse pharmacological properties. These compounds exhibit potent antioxidant, anti-inflammatory, antimicrobial, and anticancer effects, making mangosteen a valuable natural product for medicinal and nutraceutical applications (Mahmudah et al. 2020). α-mangostin, the primary xanthone in the pericarp, has been shown to play a significant role in promoting apoptosis in cancer cells, while also offering protection against oxidative stress and inflammation (Plaza et al. 2021). Xanthones, particularly those derived from *Garcinia mangostana*, have shown remarkable anticancer properties through various mechanisms. Studies have demonstrated that α-mangostin, a major xanthone, induces apoptosis and inhibits proliferation in colon and breast cancer cells (Pedraza-Chaverri et al. 2008). Additionally, xanthones target mitochondria and modulate cell cycle regulators, such as p21 and cyclin D1, to suppress cancer cell growth (Li et al. 2014). Furthermore, xanthones inhibit metastasis by suppressing epithelial-mesenchymal transition (EMT] in breast cancer cells (Li et al. 2014). In vivo studies have also confirmed their antitumor activity, with α-mangostin reducing tumor size and angiogenesis in skin cancer models (Kritsanawong et al. 2016). Lastly, the antioxidant properties of xanthones contribute to their anticancer effects by protecting cells from oxidative stress (Jung et al. 2006).

The chemical complexity of mangosteen and the high concentration of xanthones in the pericarp underscore the importance of optimizing extraction techniques to isolate these bioactive compounds. Emerging methods such as ultrasound-assisted extraction (UAE) and the use of natural deep eutectic solvents (NaDES) have been identified as sustainable and efficient alternatives to traditional solvent-based extraction processes. These advanced methods not only increase extraction efficiency but also reduce environmental impact, further enhancing the viability of mangosteen for industrial applications (Plaza et al. 2021).

Table 1 highlights the primary bioactive compounds present in mangosteen. Among these, α-mangostin (1,3,6-trihydroxy-7-methoxy-2,8-bis(3-methyl-2-butenyl)-9H-xanthen-9-one), a hydrophobic polyphenol, is one of the most significant xanthone derivatives in the fruit, playing a crucial role in its potent antioxidant properties (Guo et al. 2016). In the pericarp, α-mangostin constitutes approximately 69.1%, making it the dominant xanthone present (Wittenauer et al. 2012). This high concentration contributes to the fruit's notable therapeutic effects, particularly its antioxidant, anti-inflammatory, and anticancer properties.

**Table 1:** Main bioactive compounds of mangosteen *[Garcinia mangostana L] [14; 15]*

Phenolic acids	Flavonoids	Tannins	Xanthones
Chlorogenic acid, vinyl acid protocatechin acid p-coumaric acid Ferulic acid, gallic acid, caffeic acid, hydroxybenzoic	Epicatechin Porcatechin, cyanidin 3-sophoroside, cyanidin 3-glucoside, myricetin, rutin Quercetin	Procyanidin	α-mangostin ß-mangostin γ-mangostin Rubraxanthone 9-Hydroxycalabaxanthone Tetrahydroxyxanthone Garcixanthone A Garcixanthone B Garcixanthone C Euxanthone Garmoxanthone Mangostanazanthone III/VIII Mangostanaxanthone IV/VII Tovophylin A gartanin 8-Deoxygartanin Garcinone C Garcinone E Garcinone E

Following α-mangostin, γ-mangostin is the second most abundant compound in the mangosteen pericarp, accounting for 17.6% of the xanthone content. γ-mangostin has also demonstrated significant biological activities, including anti-inflammatory and anti-tumor effects. Other bioactive compounds, making up the remaining 13.3%, include gartanin, 8-deoxygartanin, garcinone E, and 1,3,7-trihydroxy-2,8-di-(3-methylbut-2-enyl) xanthone (Wittenauer et al. 2012); Each of these compounds contributes to the overall pharmacological profile of mangosteen, offering a wide range of health benefits, including antimicrobial, hepatoprotective, and neuroprotective effects (Plaza et al. 2021). These xanthones, particularly α- and γ-mangostin, have been the subject of numerous studies due to their potential in managing oxidative stress-related diseases, including cancer, cardiovascular diseases, and neurodegenerative conditions. As research continues to uncover the therapeutic potential of these compounds, mangosteen's relevance in the nutraceutical and pharmaceutical industries continues to grow (Plaza et al. 2021).

### Pericarp

*Garcinia mangostana* L. pericarp and exocarp are rich in various bioactive compounds, including phenolic acids, anthocyanins, isoprenylated xanthone derivatives, and procyanidins. Phenolic acids are well-known for their antioxidant properties, which help combat oxidative stress and inflammation (Hartigh 2019). Anthocyanins, which provide the fruit with its distinctive purple hue, also contribute to its antioxidant and anti-inflammatory effects (Oliver et al. 2020). Isoprenylated xanthones, a unique class of compounds found predominantly in mangosteen, have garnered significant interest for their diverse therapeutic potential (Zhang et al. 2019). Procyanidins, another group of flavonoids, further enhance the fruit's beneficial properties (Weiss et al. 2017).

The bioactive content of mangosteen pericarp found higher levels of total phenols and flavonoids; Pusat et al. (2023) determinate the total phenol content at 219.37 mg GAE/g FEW, and the total flavonoid content was 201.84 mg QE/g FEW. This high concentration of bioactive compounds is attributed to the rich presence of flavonoids and phenolics in *Garcinia mangostana*. The study also identified several key phenolic acids and flavonoids in the extracts, including chlorogenic acid, vinyl acid, protocatechuic acid, p-coumaric acid, ferulic acid, gallic acid, caffeic acid, epicatechin, procyanidin, cyanidin 3-sophoroside, cyanidin 3-glucoside, myricetin, rutin, and quercetin (Zarena and Sankar 2012).

Mangosteen pericarp is particularly noted for its high content of prenylated xanthones, which have shown a wide range of biological activities. These xanthones are potent antioxidants and possess anticancer, anti-inflammatory, and antiproliferative properties (Musso et al. 2018; Oh et al. 2020). For example, α-mangostin, one of the most studied xanthones, has demonstrated efficacy against various cancer cell lines, including those associated with cervical, gastric, colorectal, hepatocellular, and breast cancers (Williams and Downes 2017). Research indicates that α-mangostin can significantly reduce multicellular tumor spheroids derived from breast cancer cell Lines at a concentration of 30 μg/mL (Kiso et al. 2019).

Other notable bioactive xanthones from mangosteen include garcinone E and gartanin. Garcinone E has shown promise in inhibiting ovarian cancer cells (Zhang et al. 2018), while gartanin is effective against cervical cancer cell lines. Gartanin has demonstrated cytotoxic effects against cervical cell lines, inducing apoptosis through caspase activation and downregulation of antiapoptotic proteins (Akao et al. 2008). Additionally, β-mangostin is recognized for its antibacterial and anti-inflammatory properties, and it has been investigated for its potential anti-carcinogenic effects, particularly against hepatocellular carcinoma (Jefferson et al. 2018).

These findings underscore the therapeutic potential of mangosteen extracts in treating various health conditions and highlight the fruit's role as a valuable source of bioactive compounds.

### Seeds

*Garcinia mangostana* L. seeds contain flavonoids and xanthones during their development and germination phases. These compounds serve as a defensive strategy to protect the seeds' viability throughout their growth. According to Noor et al. (2016), mangosteen seeds contain significantly higher levels of these compounds compared to other Garcinia species, such as Garcinia atroviridis, Garcinia shoulderniana, and Garcinia prainiana. The oil content of mangosteen seeds ranges from approximately 21–28% of their dry weight. In addition to starch and oil, mangosteen seeds contain sugars (e.g., glucose, fructose), amino acids (e.g., phenylalanine, tyrosine), lignin, and tannins (Williams and Downes 2017; Kiso et al. 2019). They also exhibit high total phenolic (TPC) and flavonoid (TFC) content, primarily due to compounds like xanthones and tannins. These bioactive components contribute to the seeds' strong antioxidant properties, offering protective effects against oxidative stress and potential health benefits.

### Aril (pulp)

The edible part of *Garcinia mangostana* L. contains a lower concentration of phytochemical compounds, such as phenolic acids and flavonoids, compared to the peel, rind, and seeds. Agrawal et al. (Patil et al. 2021) conducted a comprehensive analysis to measure and compare the total phenolic content (TPC), total flavonoid content (TFC), and antioxidant activity across different parts of the fruit. Their study revealed that the TPC in the edible aril was significantly lower at 2.64 μg/mL, compared to 8.56 μg/mL in the pericarp. Similarly, the TFC was also lower in the edible aril at 6.56 μg/mL, while the pericarp exhibited a higher TFC of 9.64 μg/mL. This discrepancy underscores the higher concentration of these bioactive compounds in the non-edible parts of the fruit.

Conversely, Zadernowski et al. (2009) reported that the yield of crude phenol extract from the aril was 6.8%, which is notably higher than the 5.8% yield from the peel. This finding suggests that the aril, despite having lower concentrations of phenolic compounds compared to the pericarp, still contributes significantly to the overall phenolic content of the fruit. The difference in extraction yields highlights the complex distribution of phenolic compounds within the different parts of the fruit.

In addition to phenolic acids, Vien et al. (2021) identified ten distinct phenolic acids present in mangosteen. Among these, p-hydroxybenzoic acid was found to be the predominant phenolic acid in the aril. The xanthone content in the aril includes several important compounds such as γ-mangostin, 8-deoxygartanin, 1,3,8-trihydroxy-4-methylxanthone, gartanin, α-mangostin, and garcinone E (Jung et al. 2006). These xanthones are known for their various health benefits, including antioxidant and anti-inflammatory properties.

## Extraction technologies: current advances and techniques

For many years, there has been significant interest in obtaining bioactive compounds from various plant sources, along with the development and application of extraction methods. Traditional extraction techniques such as maceration, Soxhlet extraction, and hydrodistillation have been widely used (Ortíz Aguilar et al. 2016). However, these conventional methods often require lengthy processing times and substantial amounts of solvents, which can be inefficient and environmentally taxing (Chhouk et al. 2016).

To address these limitations, more advanced extraction technologies have been proposed and investigated. Techniques such as microwave-assisted extraction (MAE) (Rodsamran and Sothornvit 2019), ultrasound-assisted extraction (UAE) (Tiwari 2015), and supercritical fluid extraction (SFE) (Ghasemzadeh et al. 2018). have emerged as promising alternatives. MAE uses microwave energy to heat solvents rapidly and uniformly, enhancing the extraction efficiency and reducing processing time. UAE employs ultrasonic waves to create cavitation bubbles in the solvent, improving the extraction of bioactive compounds. SFE utilizes supercritical fluids, such as carbon dioxide, to extract compounds under high pressure and temperature, offering high selectivity and reducing the need for toxic chemical solvents. In the context of obtaining bioactive compounds from plant sources, both conventional methods and modern extraction technologies are utilized. In Table 2 outlines the different extraction techniques, emphasizing their respective benefits such as efficiency, yield, and ease of application, as well as potential drawbacks such as cost, time consumption, and the use of harmful solvents. Table 3, on the other hand, provides a detailed comparison of the outcomes achieved using these methods, specifically focusing on the types and quantities of bioactive compounds extracted from Mangosteen, such as xanthones, flavonoids, and others.

**Table 2:** Advantages and disadvantages of extraction methods

Extraction method	Advantages	Disadvantages	Reference
Solvent extraction	Low cost, easy to implement, widely used	Use of toxic solvents, low selectivity, chemical residues	Azmir et al. (2013)
Hot water extraction	Safe, cost-effective, no organic solvents required	Low efficiency for non-polar compounds, high temperature may degrade compounds	Plaza and Turner (2015)
Supercritical CO_2_ extraction	High selectivity, no toxic residues, suitable for thermolabile compounds	High cost, requires specialized equipment	Yuvanatemiya et al. (2022)
Ultrasound-assisted extraction	Fast, efficient, enhances compound release	May degrade sensitive compounds, requires constant energy input	Chemat et al. (2017a)
Microwave-assisted extraction	Fast, efficient, reduced solvent use	High initial cost, risk of overheating	Chemat et al. (2017a)
Pressurized liquid extraction [PLE]	High efficiency, fast, reduced solvent use	High cost, requires specialized equipment	Mustafa and Turner (2011)
Enzymatic extraction	Selective, mild conditions, environmentally friendly	Enzyme cost, longer processing time	Puri et al. (2012)

**Table 3:** Literature results on extraction of bioactive compounds from mangosteen

Extraction method	Compounds extracted	Optimal conditions	Yield	References
Solvent extraction	Xanthones [α-mangostin, γ-mangostin]	70% ethanol, 60 °C, 2 h	85–90% xanthone recovery	Zadernowski et al. (2009)
Supercritical CO_2_ extraction	Xanthones, polyphenols	40 °C, 300 bar, 2 h	95% xanthone recovery	Yuvanatemiya et al. (2022)
Ultrasound-assisted extraction	Xanthones, flavonoids	50% ethanol, 40 °C, 30 min	92% xanthone recovery	Chemat et al. (2017b)
Microwave-assisted extraction	Xanthones, tannins	Water, 100 °C, 10 min	88% xanthone recovery	Chemat et al. (2017a)
Pressurized liquid extraction [PLE]	Xanthones, polyphenols	50% ethanol, 100 °C, 20 min	94% xanthone recovery	Mustafa and Turner (2011)
ENZYMATIC Extraction	Xanthones, polysaccharides	Cellulase, 50 °C, pH 5, 4 h	80% xanthone recovery	Puri et al. (2012)

### Conventional extraction technique

Conventional extraction, commonly referred to as solid–liquid extraction [SLE], remains one of the most widely employed techniques for isolating bioactive compounds from various plant sources (Barba et al. 2016a). This method relies on the extraction capacity of different solvents combined with the application of heat or agitation to facilitate the release of compounds from solid plant materials into a liquid phase (Jha and Sit 2022). The efficiency of SLE depends on several factors, including the choice of solvent, extraction time, temperature, and agitation speed. Solvents are selected based on their ability to dissolve the target compounds effectively, and adjustments in temperature and agitation can enhance the extraction process by increasing the solubility of the compounds and improving their release from the plant matrix (Barba et al. 2016a).

#### Maceration

Maceration is a widely used extraction technique that operates at room temperature (Jha and Sit 2022). This process involves grinding plant material to increase its surface area, which enhances the interaction between the material and the solvent [known as the menstruum]. By doing so, the extraction of bioactive compounds becomes more efficient. Once the solute and solvent are mixed, unit operations such as filtering and pressing are employed to isolate the desired extract. Maceration is advantageous due to its low investment costs and the ability to modulate selectivity based on the choice of solvent (Renard 2018).

Using the maceration technique, Pothitirat et al. (Pothitirat et al. 2010a) prepared 95% ethanolic extracts of mangosteen peel at room temperature for a duration of 10 days. This procedure resulted in an extract yield of 24.04% by dry weight. The extract had an α-mangostin content of 13.32%, a total phenolic content of 24.31 g GAE/100 g extract, and a Tannin content of 39.52 g TAE/100 g extract. These findings indicate a significant presence of bioactive compounds in the mangosteen peel. In contrast, Naczk et al. (2011) reported that a crude extract from the mangosteen aril, using 70% aqueous ethanol, had a yield of 6.8% and a total phenol content of 9.3 g GAE/100 g. This data highlights that the phenolic content of the mangosteen peel extract is notably higher than that of the edible part of the fruit, demonstrating the peel's greater richness in bioactive compounds.

#### Soxhlet extraction

Soxhlet extraction is a standard technique often used as a benchmark for evaluating the efficiency of other solid–liquid extraction methods (Barba et al. 2016b). This method involves continuously extracting an active component from a solid mixture by using a solvent. Soxhlet extraction is favored for its simplicity and applicability at elevated temperatures, which enhances the extraction kinetics. It also offers the advantages of low initial costs, eliminates the need for filtration during the extraction process, and ensures constant contact between the solvent and the sample throughout the extraction (Jha and Sit 2022).

According to Santong-aun et al. (Assawarachan et al. 2011), the Soxhlet extraction (SE) method requires approximately 15 h to extract compounds from mangosteen peel using 95% ethanol. In their study, α-mangostin was extracted for 15 h at a temperature range of 65–70 °C. The results indicated a yield of 25.45% ± 0.22% w/w of dry powder; however, the percentage of α-mangostin obtained was relatively low compared to yields from other extraction methods assessed in the same study, such as MAE took a shorter extraction time, and the higher α-mangostin content (49.79 ± 0.15% w/w of crude extract) than shaking-water-bath extraction SWE (45.83 ± 0.02% w/w of crude extract) and SE (34.82 ± 0.17% w/w of crude extract).

Further research by Yoswathana and Eshtiaghi (2015) demonstrated that the extraction time significantly impacts the yield of α-mangostin. Their findings showed that increasing the extraction time from 0.5 to 2 h resulted in a recovery of 41.41 mg/g of α-mangostin from the dry sample, highlighting the importance of optimizing extraction duration for maximizing yield. Additionally, Pothitirat et al. (2010b) reported a dry weight yield of 26.60 using 95% ethanol for Soxhlet extraction, with a total phenolic content of 24.83 g GAE/100 g. In contrast, when the ethanol concentration was reduced to 70%, the extract yield increased to 27.70%, and the total phenolic content rose to 26.88 g GAE/100 g. These variations illustrate how changes in solvent concentration can affect both the yield and phenolic content of the extract.

#### Hydrodistillation

Hydrodistillation is one of the oldest and still widely used extraction techniques for isolating bioactive compounds and essential oils from plant materials and fruit waste (Jha and Sit 2022). Unlike other methods, hydrodistillation does not rely on organic solvents and can be performed either before or after the dehydration of plant materials. There are three primary types of hydrodistillation: water distillation, water and steam distillation, and direct steam distillation.

In water distillation, plant materials are packed into a still, and water is added and brought to a boil. Alternatively, in direct steam distillation, hot steam is injected directly into the plant material. Both hot water and steam play crucial roles in facilitating the release of bioactive compounds from plant tissues (Azmir et al. 2013). This method provides several advantages over techniques like Soxhlet extraction and maceration, including the elimination of organic solvents, reduced need for pre-treatment of plant materials, and faster extraction times (Jha and Sit 2022). Hydrodistillation is particularly effective for extracting essential oils from parts of *Garcinia mangostana*, such as the leaf and bark. This technique is widely used for extracting essential oils from various plant parts, including the leaf and bark, which are often rich in bioactive compounds with numerous therapeutic properties. For example, hydrodistillation of *Cinnamomum verum* [cinnamon] bark yields essential oils rich in cinnamaldehyde, known for its antimicrobial and anti-inflammatory effects (Alizadeh Behbahani et al. 2020). Similarly, the leaves of *Eucalyptus globulus* produce essential oils rich in eucalyptol, a compound with well-known antiseptic and expectorant properties (Almas et al. 2021). Additionally, the leaves of *Mentha piperita* [peppermint] contain essential oils high in menthol, which are valued for their cooling sensation and digestive benefits (McKay and Blumberg 2006). These examples demonstrate the versatility of hydrodistillation in extracting essential oils from both the leaf and bark of various plants, each offering a unique set of bioactive compounds. The efficiency of hydrodistillation, like other extraction methods, is influenced by various factors including the choice of solvents, the molecular affinity between the solvent and solute, mass transfer dynamics, and the use of co-solvents. Additionally, considerations for environmental safety and toxicity are crucial for ensuring the method's feasibility both from human and economic perspectives.

### Microwave assisted extraction (MAE)

MAE is an advanced extraction technique that utilizes volumetric heating to efficiently extract desired compounds from natural sources (Ali et al. 2019). This method involves using microwave energy to heat polar solvents that are in contact with solid samples. The microwave irradiation facilitates the partitioning of compounds of interest between the sample and the solvent, leading to reduced extraction times, lower solvent consumption, and rapid energy transfer. The volumetric heating allows for effective diffusion of the solvent within the extraction medium, enhancing the overall efficiency of the process (Dahmoune et al. 2015). As a result, MAE often produces higher extraction rates and more favorable outcomes in terms of cost-effectiveness. MAE has proven to be a highly effective alternative for extracting phytocompounds from mangosteen, particularly α-mangostin from the fruit’s pericarp (Hiew et al. 2021). For example, MAE has been successfully used to extract xanthones from *Garcinia mangostana* (mangosteen), yielding high concentrations of bioactive compounds with antioxidant and anti-inflammatory properties (Mohammad et al. 2019). Similarly, MAE has been applied to extract polyphenolic compounds from *Hibiscus sabdariffa* (roselle), enhancing the yield of anthocyanins and other antioxidants (Pimentel-Moral et al. 2018). Additionally, MAE has been employed to extract essential oils and other bioactive compounds from *Cinnamomum zeylanicum* (cinnamon) leaves, improving the recovery of antioxidant compounds (Kallel et al. 2019). However, it is important to note that polyphenolic compounds, including xanthones and flavonoids, are generally soluble in organic solvents and may require nonpolar solvents for optimal extraction and dissolution (Aizat et al. 2019c). Ghasemzadeh et al. (2017) optimized a microwave-assisted protocol for extracting α-mangostin using ethyl acetate as a green solvent. Their study achieved a concentration of α-mangostin of 121.01 mg/g of dry matter within just 3.16 min, utilizing a microwave power of 189.20 W and a solvent extraction percentage of 70.20% v/v. Among the optimized extracts, trans-ferulic acid and catechin were identified as the most abundant compounds.

Similarly, Hasan et al. (2016) reported a flavonoid content of 11.82% in extracts obtained within 32.9 min using 70% ethanol. This highlights the efficiency of MAE in extracting flavonoids and other bioactive compounds. MAE has been successfully used to extract xanthones from *Garcinia mangostana* pericarp, under optimal conditions, including a microwave power of 600 W, extraction time of 5 min, and solvent concentration of 60% ethanol. This process yields high concentrations of bioactive compounds, including catechin and quercetin, which are the primary flavonoids present in mangosteen pericarp, with antioxidant and anti-inflammatory properties (Mohammad et al. 2019). Additionally, MAE can also be employed to extract other valuable compounds from mangosteen pericarp, including pectin, further demonstrating its versatility and effectiveness in obtaining a range of technological compounds (Maulion and v. 2022).

### Ultrasound (UAE)

UAE is an emerging green technology with significant potential across various technological processes (Moreira et al. 2019). This technique operates on principles similar to sound waves but at ultrasonic frequencies. Ultrasound waves generate a series of compression and rarefaction cycles that propagate through the medium, creating mechanical forces within the liquid (Vinatoru et al. 2017). When applied as an extraction method, ultrasound offers several advantages over conventional techniques, including reduced extraction times, lower temperatures, and higher yields (Moreira et al. 2019). The effectiveness of ultrasound in enhancing extraction efficiency is attributed to its ability to generate cavitation bubbles within the liquid. As these bubbles collapse, they create microjets that disrupt plant cell walls, facilitating the release of bioactive compounds (Cheok et al. 2012). UAE has been particularly effective in extracting phenolic compounds, anthocyanins, and flavonoids from mangosteen peel and pulp (Kallel et al. 2019). For instance, Muzykiewicz et al. (2020) investigated the antioxidant activity and polyphenol content in extracts from various edible parts of fresh *Garcinia mangostana* using UAE. The researchers evaluated the antioxidant activity and polyphenol content in extracts from various edible parts of fresh *Garcinia mangostana* (mangosteen) using Ultrasound-Assisted Extraction (UAE). They employed ethanol at varying concentrations (20%, 40%, 70%, and 96% v/v) and extraction times (15, 30, and 60 min). The results indicated that UAE, particularly with extraction times of 30 or 60 min and using ethanol concentrations higher than 20% (v/v), was an effective method for obtaining bioactive compounds. These conditions significantly enhanced the antioxidant activity and polyphenol content, demonstrating the effectiveness of UAE for extracting polyphenols from the pericarp and pulp of mangosteen, thus highlighting its potential for improving the bioactive profile of mangosteen extracts.

Furthermore, Suryono et al. (Suryono et al. 2019) explored the impact of frequency, temperature, and sonication time on the xanthone content of mangosteen peel extracts. The findings demonstrated that Ultrasound-Assisted Extraction (UAE) outperformed conventional methods, with optimal extraction conditions achieved at a frequency of 40 kHz, a temperature of 35 °C, and a sonication time of 30 min. Under these conditions, the xanthone content in the mangosteen peel extract reached 93 ppm. In comparison, conventional extraction methods yielded a significantly lower xanthone content at the same frequency, temperature, and sonication time. This study highlights the superior performance of UAE in extracting valuable bioactive compounds, emphasizing its advantages over traditional extraction techniques. Overall, UAE proves to be a highly efficient and environmentally friendly method for extracting bioactive compounds from plant sources, including mangosteen, by enhancing yields and reducing processing times.

### Supercritical fluid-assisted extraction [SFE]

SFE is an advanced green technology that has continuously evolved to enhance the separation of bioactive compounds from natural materials. This technique utilizes supercritical fluids, which possess both liquid and gaseous properties when subjected to temperatures and pressures above their critical points. Due to their relatively low viscosities and high diffusivities, supercritical fluids can significantly improve diffusion and mass transfer, thereby reducing extraction times (Uwineza and Waśkiewicz 2020). Additionally, the density of supercritical fluids is higher than that of gases but similar to that of liquids, which enhances their solvation power.

Among the various supercritical fluids, supercritical carbon dioxide (CO_2_) is the most commonly employed solvent for extracting bioactive compounds from plant materials (Capuzzo et al. 2013). CO_2_ is favored because it is non-toxic, environmentally friendly, and has a relatively low critical pressure (73.8 bar) and temperature (31.2 °C). These properties prevent oxidation of the extracts and facilitate easy separation of the extract from the solvent due to CO_2_^’^s volatility. Furthermore, CO_2_ is cost-effective, colorless, and odorless. To enhance the extraction of polar compounds, cosolvents such as ethanol are often used in combination with supercritical CO_2_. Ethanol is particularly effective due to its low boiling point and its ability to be easily separated from the final extract (Moreira et al. 2019).

SFE technology offers a promising alternative for the extraction of α- and γ-mangostin from mangosteen peel. For example, in a study by Lee et al. (2019) SFE was employed to extract α-mangostin from mangosteen pericarp using supercritical CO₂ as the solvent. The extraction conditions included pressures ranging from 30 to 40 MPa, a temperature of 60 °C, and a CO₂ flow rate of 1.7 g/min, with the addition of virgin coconut oil (VCO) as a co-extractant at concentrations ranging from 0 to 20%. The results showed that both the mangosteen pericarp extract (MPE) yield and the extraction efficiency increased with the amount of co-extractant added. The highest yield of MPE (17%) was achieved at a pressure of 35 MPa and 20% VCO. Furthermore, the scCO₂-extracted MPE exhibited a higher bio-accessibility of α-mangostin (91%) compared to the α-mangostin extracted using ethanol (30%) or hexane (50%). This study highlights that the combination of supercritical CO₂ extraction with virgin coconut oil as a co-extractant provides an efficient method for producing high bio-accessible α-mangostin from mangosteen pericarp. Thus, supercritical CO_2_ extraction used with virgin coconut oil co-extractant provides a method for producing high bio-accessible α-mangostin from mangosteen pericarp extract. The process was also eco-friendly, as it avoids the use of toxic solvents typically associated with conventional extraction methods. This method is efficient in isolating xanthones without requiring large volumes of chemicals or complex equipment. For instance, Chhouk et al. (2016) investigated the use of a hydrothermal process combined with supercritical CO_2_ for extracting bioactive compounds from mangosteen pericarp. Their study achieved an optimal extraction yield of 39.9% w/w. These studies demonstrate the effectiveness of SFE and its variations in optimizing the extraction of valuable compounds from mangosteen.

### Enzymatic extraction of *Garcinia mangostana*

Enzymatic extraction is an efficient and eco-friendly method employed to recover bioactive compounds from *Garcinia mangostana*, commonly known as mangosteen, which is renowned for its rich content of xanthones, polyphenols, and antioxidants. This technique involves the use of specific enzymes to break down plant cell walls, facilitating the release of these bioactive compounds into the extraction solvent (Han et al. 2024) Mechanism of Enzymatic Extraction: Enzymatic extraction typically utilizes cellulase, pectinase, or hemicellulase, which target the structural polysaccharides like cellulose, hemicellulose, and pectin in the cell wall. These enzymes effectively degrade the cell wall matrix, releasing the valuable phytochemicals present in the mangosteen pericarp [outer rind] and pulp. The process can be optimized by adjusting factors such as enzyme concentration, temperature, pH, and extraction time, to maximize the yield of desired bioactive compounds (Tan et al. 2023). Advantages of Enzymatic Extraction. Higher Yield: Enzyme-assisted extraction can enhance the recovery of bioactive compounds, particularly those that are difficult to extract using conventional methods such as solvent extraction or maceration. Selective Extraction: Enzymatic methods allow for the selective extraction of specific compounds, improving the purity of the final extract (Jung et al. 2006). Eco-friendly: This method reduces the use of organic solvents, making it a more sustainable and environmentally friendly option for bioactive compound recovery (Zamarudin et al. 2023). Mangosteen has garnered interest due to its high antioxidant potential, attributed primarily to the presence of xanthones such as alpha-mangostin. Enzymatic extraction allows for the isolation of these xanthones, which are known for their anti-inflammatory, anticancer, and antimicrobial properties. The extracted compounds have applications in the food, pharmaceutical, and cosmetic industries (Zamarudin et al. 2023). A study by Moh Moh Han., et al. (2024) demonstrated that cellulase and pectinase enzymes were used to extract xanthones from mangosteen rind, yielding higher amounts of antioxidant compounds compared to traditional solvent-based methods. The study showed that optimizing enzyme concentration and extraction time led to a significant improvement in both the extraction yield and the antioxidant activity of the final extract.

## Conclusions

*Garcinia mangostana* L. is a rich source of biologically active substances, including phenolic compounds, anthocyanins, flavonoids, and isoprenylated xanthone derivatives, with α-mangostin being the principal compound known for its potent antioxidant properties. These bioactive compounds offer significant potential for applications in the development of functional foods and pharmaceutical products, thanks to their diverse health benefits. Conventional extraction techniques, such as maceration, Soxhlet extraction, and hydrodistillation, have demonstrated effectiveness in isolating bioactive compounds from mangosteen. Despite their efficiency, these methods have notable drawbacks, including lengthy extraction times and substantial solvent usage. Nevertheless, their low initial investment costs and simplicity have maintained their relevance in many laboratories and industries. In recent years, there has been growing interest in advanced extraction technologies that address some of the limitations of conventional methods. Techniques such as MAE, UAE, and SFE offer several advantages, including reduced extraction times, lower solvent consumption, and enhanced extraction yields. These methods are considered more environmentally friendly and cost-effective in the long run, owing to their ability to minimize the use of toxic solvents and reduce overall processing time. Overall, while conventional methods remain widely used due to their practicality and cost-effectiveness, the integration of advanced extraction technologies presents a promising direction for optimizing the extraction of valuable bioactive compounds from mangosteen. These newer technologies not only improve the efficiency and sustainability of the extraction process but also hold the potential for enhancing the quality and yield of bioactive compounds, paving the way for more innovative applications in the food and pharmaceutical industries.

## Data Availability

Not applicable.

## References

[CR1] Abdul-Rahman A, Goh HH, Loke KK, Noor NM, Aizat WM (2017) RNA-seq analysis of mangosteen [*Garcinia mangostana* L.] fruit ripening. Genom Data 12:159–160 28560167 10.1016/j.gdata.2017.05.013 PMC5435573

[CR2] Aizat WM, Ahmad-Hashim FH, Syed Jaafar SN (2019a) Valorization of mangosteen, “The Queen of Fruits”, and new advances in postharvest and in food and engineering applications: a review. J Adv Res 20:61–70 31210985 10.1016/j.jare.2019.05.005 PMC6562293

[CR3] Aizat WM, Ahmad-Hashim FH, Syed Jaafar SN (2019b) Valorization of mangosteen, “The Queen of Fruits”, and new advances in postharvest and in food and engineering applications: a review. J Adv Res 20:61–70 31210985 10.1016/j.jare.2019.05.005 PMC6562293

[CR4] Aizat WM, Jamil IN, Ahmad-Hashim FH, Noor NM (2019c) Recent updates on metabolite composition and medicinal benefits of mangosteen plant. PeerJ. 10.7717/peerj.6324 30755827 10.7717/peerj.6324 PMC6368837

[CR5] Akao Y, Nakagawa Y, Iinuma M, Nozawa Y (2008) Anti-cancer effects of xanthones from pericarps of mangosteen. Int J Mol Sci 9:355–370 19325754 10.3390/ijms9030355 PMC2635669

[CR6] Ali A, Chua BL, Chow YH (2019) An insight into the extraction and fractionation technologies of the essential oils and bioactive compounds in *Rosmarinus officinalis* L.: past, present and future. TrAC Trends Analyt Chem 118:338–351

[CR7] Alizadeh Behbahani B, Falah F, Lavi Arab F, Vasiee M, Tabatabaee YF (2020) Chemical composition and antioxidant, antimicrobial, and antiproliferative activities of cinnamomum zeylanicum bark essential oil. Evidence-Based Complement Alternat Medic 2020(1):5190603. 10.1155/2020/5190603 10.1155/2020/5190603 PMC7210559 32419807

[CR8] Almas I, Innocent E, Machumi F, Kisinza W (2021) Chemical composition of essential oils from eucalyptus globulus and eucalyptus maculata grown in Tanzania. Sci Afr 1(12):e00758

[CR9] Assawarachan R, Noomhorm A, Satong-Aun W, Assawarachan R, Noomhorm A (2011) The influence of drying temperature and extraction methods on α-mangostin in mangosteen pericarp. J Food Sci Eng 1:85

[CR10] Azmir J, Zaidul ISM, Rahman MM, Sharif KM, Mohamed A, Sahena F et al (2013) Techniques for extraction of bioactive compounds from plant materials: a review. J Food Eng 117(4):426–436

[CR11] Barba FJ, Zhu Z, Koubaa M, Sant’Ana AS, Orlien V (2016) Green alternative methods for the extraction of antioxidant bioactive compounds from winery wastes and by-products: a review. Trends Food Sci Technol 1:96–109

[CR12] Binti Zamarudin Z, Bin Abdullah Sani MS, Nordin NF, Amid A, Hashim AM (2023) Mangosteen [*Garcinia mangostana*]: extraction, purification, bioactivities and toxicities. Halalsphere 3(2):13–27

[CR13] Capuzzo A, Maffei ME, Occhipinti A (2013) Supercritical fluid extraction of plant flavors and fragrances. Molecules 18(6):7194 23783457 10.3390/molecules18067194 PMC6270407

[CR14] Chemat F, Rombaut N, Sicaire AG, Meullemiestre A, Fabiano-Tixier AS, Abert-Vian M (2017a) Ultrasound assisted extraction of food and natural products. Mechanisms, techniques, combinations, protocols and applications. A review. Ultrason Sonochem 34:540–560. 10.1016/j.ultsonch.2016.06.035 27773280 10.1016/j.ultsonch.2016.06.035

[CR15] Chemat F, Rombaut N, Sicaire AG, Meullemiestre A, Fabiano-Tixier AS, Abert-Vian M (2017b) Ultrasound assisted extraction of food and natural products. Mechanisms, techniques, combinations, protocols and applications. A review. Ultrason Sonochem 34:540–560. 10.1016/j.ultsonch.2016.06.035 27773280 10.1016/j.ultsonch.2016.06.035

[CR16] Cheok CY, Chin NL, Yusof YA, Talib RA, Law CL (2012) Optimization of total phenolic content extracted from *Garcinia mangostana* Linn. hull using response surface methodology versus artificial neural network. Ind Crops Prod 40(1):247–253

[CR17] Chhouk K, Quitain AT, Gaspillo PAD, Maridable JB, Sasaki M, Shimoyama Y et al (2016) Supercritical carbon dioxide-mediated hydrothermal extraction of bioactive compounds from *Garcinia Mangostana* pericarp. J Supercrit Fluids 1(110):167–175

[CR18] Dahmoune F, Nayak B, Moussi K, Remini H, Madani K (2015) Optimization of microwave-assisted extraction of polyphenols from *Myrtus communis* L. leaves. Food Chem 166:585–595 25053097 10.1016/j.foodchem.2014.06.066

[CR19] de Mello RFA, de Souza Pinheiro WB, Benjamim JKF, de Siqueira FC, Chisté RC, Santos AS (2021) A fast and efficient preparative method for separation and purification of main bioactive xanthones from the waste of Garcinia mangostana L by high-speed countercurrent chromatography. Arabian J Chem 14(8):103252

[CR20] den Hartigh LJ (2019) Conjugated linoleic acid effects on cancer, obesity, and atherosclerosis: a review of pre-clinical and human trials with current perspectives. Nutrients 11(2):370 30754681 10.3390/nu11020370 PMC6413010

[CR21] Ghasemzadeh A, Jaafar HZE, Rahmat A, Swamy MK (2017) Optimization of microwave-assisted extraction of zerumbone from Zingiber zerumbet L rhizome and evaluation of antiproliferative activity of optimized extracts. Chem Cent J 11(1):5 28123448 10.1186/s13065-016-0235-3 PMC5216017

[CR22] Ghasemzadeh A, Jaafar HZE, Baghdadi A, Tayebi-Meigooni A (2018) Alpha-mangostin-rich extracts from mangosteen pericarp: optimization of green extraction protocol and evaluation of biological activity. Molecules 23(8):1852 30044450 10.3390/molecules23081852 PMC6222712

[CR23] Guo M, Wang X, Lu X, Wang H, Brodelius PE (2016) α-Mangostin extraction from the native mangosteen (*Garcinia mangostana* L.) and the binding mechanisms of α-mangostin to HSA or TRF. PLoS ONE 11(9):e0161566. 10.1371/journal.pone.0161566 27584012 10.1371/journal.pone.0161566 PMC5008840

[CR24] Han MM, Tangpromphan P, Kaewchada A, Jaree A (2024) Recovery and partial isolation of ⍺-mangostin from mangosteen pericarpsvia sequential extraction and precipitation. PLoS ONE 19(10):e0310453 39453921 10.1371/journal.pone.0310453 PMC11508469

[CR25] Hasan AEZ, Nashrianto H, Juhaeni RN, Artika IM. (2016) Optimization of conditions for flavonoids extraction from mangosteen [*Garcinia mangostana* L.]. [cited 2022 Aug 24]; Available from: www.scholarsresearchlibrary.com

[CR26] Hiew CW, Lee LJ, Junus S, Tan YN, Chai TT, Ee KY (2021) Optimization of microwave-assisted extraction and the effect of microencapsulation on mangosteen [*Garcinia mangostana* L.] rind extract. Food Sci Technol 42:e35521

[CR27] Ibrahim MY, Hashim NM, Mariod AA, Mohan S, Abdulla MA, Abdelwahab SI et al (2016) α-Mangostin from *Garcinia mangostana* Linn: an updated review of its pharmacological properties. Arab J Chem 9(3):317–329

[CR28] Jefferson RE, Min D, Corin K, Wang JY, Bowie JU (2018) Applications of single-molecule methods to membrane protein folding studies. J Mol Biol 430(4):424 28549924 10.1016/j.jmb.2017.05.021 PMC5700846

[CR29] Jha AK, Sit N (2022) Extraction of bioactive compounds from plant materials using combination of various novel methods: a review. Trends Food Sci Technol 119(Jan 1):579–591

[CR30] Jung HA, Su BN, Keller WJ, Mehta RG, Kinghorn AD (2006) Antioxidant xanthones from the pericarp of *Garcinia mangostana* (Mangosteen). J Agric Food Chem 54(6):2077–2082 16536578 10.1021/jf052649z

[CR31] Kallel I, Hadrich B, Gargouri B, Chaabane A, Lassoued S, Gdoura R et al (2019) Optimization of cinnamon (Cinnamomum zeylanicum Blume) essential oil extraction: evaluation of antioxidant and antiproliferative effects. Evidence-Based Complement Alternat Medic 2019(1):6498347. 10.1155/2019/6498347 10.1155/2019/6498347 PMC6942840 31929818

[CR32] Kiso M, Yamayoshi S, Furusawa Y, Imai M, Kawaoka Y (2019) Treatment of highly pathogenic H7N9 virus-infected mice with baloxavir marboxil. Viruses 11(11):1066 31731678 10.3390/v11111066 PMC6893572

[CR33] Kritsanawong S, Innajak S, Imoto M, Watanapokasin R (2016) Antiproliferative and apoptosis induction of alpha-mangostin in T47D breast cancer cells. Int J Oncol 48(5):2155–2165 26892433 10.3892/ijo.2016.3399

[CR34] Lee WJ, Ng CC, Ng JS, Smith RL, Kok SL, Hee YY et al (2019) Supercritical carbon dioxide extraction of α-mangostin from mangosteen pericarp with virgin coconut oil as co-extractant and in-vitro bio-accessibility measurement. Process Biochem 1(87):213–220

[CR35] Li G, Petiwala SM, Nonn L, Johnson JJ (2014) Inhibition of CHOP accentuates the apoptotic effect of α-mangostin from the mangosteen fruit (Garcinia mangostana) in 22Rv1 prostate cancer cells. Biochem Biophys Res Commun 453(1):75–80 25261723 10.1016/j.bbrc.2014.09.054

[CR36] Mahmudah R, Adnyana IK, Sukandar EY (2020) Pharmacological effects of *Garcinia mangostana* L.: an update review. Res J Pharm Technol 13(11):5471–5476

[CR37] Maulion RV. International journal of advanced research and publications microwave assisted extraction of pectin from mangosteen [*Garcinia Mangostana*] Rind. [cited 2022 Aug 24]; Available from: www.ijarp.org

[CR38] McKay DL, Blumberg JB (2006) A review of the bioactivity and potential health benefits of peppermint tea [*Mentha piperita* L.]. Phytotherapy Res 20(8):619–633. 10.1002/ptr.1936 10.1002/ptr.1936 16767798

[CR39] Mohamed GA, Al-Abd AM, El-halawany AM, Abdallah HM, Ibrahim SRM (2017) New xanthones and cytotoxic constituents from *Garcinia mangostana* fruit hulls against human hepatocellular, breast, and colorectal cancer cell lines. J Ethnopharmacol 198:302–312 28108382 10.1016/j.jep.2017.01.030

[CR40] Mohammad NA, Abang Zaidel DN, Muhamad II, Abdul Hamid M, Yaakob H, Mohd Jusoh YM (2019) Optimization of the antioxidant-rich xanthone extract from mangosteen [*Garcinia mangostana* L.] pericarp via microwave-assisted extraction. Heliyon 5(10):e02571 31667409 10.1016/j.heliyon.2019.e02571 PMC6812211

[CR41] Mohd Noor N, Aizat WM, Hussin K, Rohani ER (2016) Seed characteristics and germination properties of four Garcinia [Clusiaceae] fruit species. EDP Sci 71(4):199–207

[CR42] Moreira SA, Alexandre EMC, Pintado M, Saraiva JA (2019) Effect of emergent non-thermal extraction technologies on bioactive individual compounds profile from different plant materials. Food Res Int 1(115):177–190 10.1016/j.foodres.2018.08.046 30599930

[CR43] Musso N, Conte L, Carloni B, Campana C, Chiusano MC, Giusti M (2018) Low-salt intake suggestions in hypertensive patients do not jeopardize urinary iodine excretion. Nutrients 10(10):1548 30347728 10.3390/nu10101548 PMC6213341

[CR44] Mustafa A, Turner C (2011) Pressurized liquid extraction as a green approach in food and herbal plants extraction: a review. Anal Chim Acta 703(1):8–18 21843670 10.1016/j.aca.2011.07.018

[CR45] Muzykiewicz A, Zielonka-Brzezicka J, Siemak J, Klimowicz A (2020) Antioxidant activity and polyphenol content in extracts from various parts of fresh and frozen mangosteen. Acta Sci Pol Technol Aliment 19(3):261–270 32978909 10.17306/J.AFS.0788

[CR46] Naczk M, Towsend M, Zadernowski R, Shahidi F (2011) Protein-binding and antioxidant potential of phenolics of mangosteen fruit [*Garcinia mangostana*]. Food Chem 128(2):292–298 25212134 10.1016/j.foodchem.2011.03.017

[CR47] Oh Y, Do HTT, Kim S, Kim YM, Chin YW, Cho J (2020) Memory-enhancing effects of mangosteen pericarp water extract through antioxidative neuroprotection and anti-apoptotic action. Antioxidants 10(1):1–23 33396950 10.3390/antiox10010034 PMC7823671

[CR48] Oliver C, Watson C, Crowley E, Gilroy M, Page D, Weber K et al (2020) Vitamin and mineral supplementation practices in preterm infants: a survey of australian and new zealand neonatal intensive and special care units. Nutrients 12(1):51 10.3390/nu12010051 PMC7019934 31878077

[CR49] Ortíz Aguilar J, Marín Mahecha O, León PA, Suarez Rivero M, Acuña Monsalve Y, Zuluaga Domínguez CM et al (2016) Potencial antioxidante de los extractos obtenidos del pericarpio y la semilla del fruto de *Garcinia mangostana* L. según el método de extracción. Agron Colomb 34(1):S709–S711

[CR50] Patil P, Agrawal M, Almelkar S, Jeengar MK, More A, Alagarasu K et al (2021) In vitro and in vivo studies reveal α-Mangostin, a xanthonoid from *Garcinia mangostana*, as a promising natural antiviral compound against chikungunya virus. Virol J 18(1):47 33639977 10.1186/s12985-021-01517-z PMC7916311

[CR51] Pedraza-Chaverri J, Cárdenas-Rodríguez N, Orozco-Ibarra M, Pérez-Rojas JM (2008) Medicinal properties of mangosteen (*Garcinia mangostana*). Food Chem Toxicol 46(10):3227–3239 18725264 10.1016/j.fct.2008.07.024

[CR52] Pimentel-Moral S, Borrás-Linares I, Lozano-Sánchez J, Arráez-Román D, Martínez-Férez A, Segura-Carretero A (2018) Microwave-assisted extraction for hibiscus sabdariffa bioactive compounds. J Pharm Biomed Anal 15(156):313–322 10.1016/j.jpba.2018.04.050 29734100

[CR53] Plaza M, Turner C (2015) Pressurized hot water extraction of bioactives. TrAC Trends Anal Chem 71(Sep 1):39–54

[CR54] Plaza M, Domínguez-Rodríguez G, Sahelices C, Marina ML (2021) A sustainable approach for extracting non-extractable phenolic compounds from mangosteen peel using ultrasound-assisted extraction and natural deep eutectic solvents. Appl Sci 11(12):5625

[CR55] Pothitirat W, Chomnawang MT, Gritsanapan W (2010a) Anti-acne-inducing bacterial activity of mangosteen fruit rind extracts. Med Princ Pract 19(4):281–286 20516704 10.1159/000312714

[CR56] Pothitirat W, Chomnawang MT, Supabphol R, Gritsanapan W (2010b) Free radical scavenging and anti-acne activities of mangosteen fruit rind extracts prepared by different extraction methods. Pharm Biol 48(2):182–186 20645837 10.3109/13880200903062671

[CR57] Puri M, Sharma D, Barrow CJ (2012) Enzyme-assisted extraction of bioactives from plants. Trends Biotechnol 30:37–44 21816495 10.1016/j.tibtech.2011.06.014

[CR58] Pusat P, Reduksi T, Bencana R, Bidang K, Pengembangan T, Alam S, et al. (2023) Tinjauan hasil-hasil penelitian tentang timbulan limbah b3 medis dan rumah tangga selama bencana pandemik Covid-19 researches review on generation of medical and municipal hazardous waste during covid-19 pandemic disaster. Vol. 4

[CR59] Renard CMGC (2018) Extraction of bioactives from fruit and vegetables: state of the art and perspectives. LWT 1(93):390–395

[CR60] Rodsamran P, Sothornvit R (2019) Extraction of phenolic compounds from lime peel waste using ultrasonic-assisted and microwave-assisted extractions. Food Biosci 28(Apr 1):66–73

[CR61] Saraswathy SUP, Lalitha LCP, Rahim S, Gopinath C, Haleema S, SarojiniAmma S et al (2022) A review on synthetic and pharmacological potential of compounds isolated from *Garcinia mangostana* Linn. Phytomedicine plus. 2(2):100253

[CR62] Suryono S, Hadiyanto H, Yasin M, Widyowati R, Muniroh M, Amalia A. (2019) Effect of frequency, temperature, and time of sonication on xanton content of mangosteen [*Garcinia mangostana* L.] peel extract through ultrasound assisted extraction. E3SWC. 125:25006. Available from: https://ui.adsabs.harvard.edu/abs/2019E3SWC.12525006S/abstract

[CR63] Tan SSY, Shanmugham M, Chin YL, An J, Chua CK, Ong ES et al (2023) Pressurized hot water extraction of mangosteen pericarp and its associated molecular signatures in endothelial cells. Antioxidants 12(11):1932 38001785 10.3390/antiox12111932 PMC10669822

[CR64] Tiwari BK (2015) Ultrasound: a clean, green extraction technology. TrAC, Trends Anal Chem 1(71):100–109

[CR65] Uwineza PA, Waśkiewicz A (2020) Recent advances in supercritical fluid extraction of natural bioactive compounds from natural plant materials. Molecules 25(17):3847 32847101 10.3390/molecules25173847 PMC7504334

[CR66] Vien LC, Chinnappan S, Mogana R (2021) Antioxidant activity of *Garcinia mangostana* L. and alpha mangostin: a review. Res J Pharm Technol 14(8):4466–4470

[CR67] Vinatoru M, Mason TJ, Calinescu I (2017) Ultrasonically assisted extraction [UAE] and microwave assisted extraction [MAE] of functional compounds from plant materials. TrAC Trends Anal Chem 97(1):159–178

[CR68] Wang A, Li D, Wang S, Zhou F, Li P, Wang Y et al (2018) γ-Mangostin, a xanthone from mangosteen, attenuates oxidative injury in liver via NRF2 and SIRT1 induction. J Funct Foods 1(40):544–553

[CR69] Weiss NH, Peasant C, Sullivan TP (2017) Intimate partner violence and HIV-risk behaviors: evaluating avoidant coping as a moderator. AIDS Behav 21(8):2233–2242 27778220 10.1007/s10461-016-1588-2 PMC5472532

[CR70] Williams H, Downes E (2017) Development of a course on complex humanitarian emergencies: preparation for the impact of climate change. J Nurs Scholarsh 49(6):661 28960808 10.1111/jnu.12339 PMC5729744

[CR71] Wittenauer J, Falk S, Schweiggert-Weisz U, Carle R (2012) Characterisation and quantification of xanthones from the aril and pericarp of mangosteens (*Garcinia mangostana* L.) and a mangosteen containing functional beverage by HPLC–DAD–MSn. Food Chem 134(1):445–452

[CR72] Yoswathana N, Eshtiaghi MN (2015) Optimization of subcritical ethanol extraction for xanthone from mangosteen pericarp. Int J Chem Eng Appl 6(2):115–119

[CR73] Yuvanatemiya V, Srean P, Klangbud WK, Venkatachalam K, Wongsa J, Parametthanuwat T et al (2022) A review of the influence of various extraction techniques and the biological effects of the xanthones from mangosteen [*Garcinia mangostana* L.] Pericarps. Molecules 27(24):8775 36557908 10.3390/molecules27248775 PMC9782657

[CR74] Zadernowski R, Czaplicki S, Naczk M (2009) Phenolic acid profiles of mangosteen fruits [*Garcinia mangostana*]. Food Chem 112(3):685–689

[CR75] Zarena AS, Sankar KU (2012) Phenolic acids, flavonoid profile and antioxidant activity in mangosteen (*Garcinia mangostana* L.) pericarp. J Food Biochem 36(5):627–633

[CR76] Zhang Y, Kabba J, Chang J, Ji W, Zhu S, Yu J et al (2018) A school-based educational intervention for school-aged children and caregivers about rational use of antibiotics in urban areas of Shaanxi province: a study protocol for a randomized controlled research. Int J Environ Res Public Health 15(9):1912 30720793 10.3390/ijerph15091912 PMC6163849

[CR77] Zhang L, Cui X, Han Y, Park KS, Gao X, Zhang X et al (2019) Hypoxic drive caused type 3 neovascularization in a preclinical model of exudative age-related macular degeneration. Hum Mol Genet 28(20):3475–3485 31518400 10.1093/hmg/ddz159 PMC7275777

